# Diagnostic accuracy of Clauss and prothrombin time–derived fibrinogen against rotational thromboelastometry FIBTEM-A5

**DOI:** 10.1016/j.rpth.2026.103439

**Published:** 2026-03-26

**Authors:** Akmez Latona, Kate Hill, Mark Rane, Alan Ho, Biswadev Mitra

**Affiliations:** 1 Emergency Department, Ipswich Hospital, Ipswich, Queensland, Australia; 2 Department of Retrieval Medicine, LifeFlight Medical, Toowoomba, Queensland, Australia; 3 School of Public Health and Preventive Medicine, Monash University, Melbourne, Victoria, Australia; 4 Faculty of Medicine, University of Queensland, Brisbane, Queensland, Australia; 5 Department of Haematology, Princess Alexandra Hospital, Woolloongabba, Queensland, Australia; 6 Pathology Queensland, Brisbane, Queensland, Australia; 7 Queensland Cyber Infrastructure Foundation, Brisbane, Queensland, Australia; 8 Emergency & Trauma Centre, Alfred Health, Melbourne, Victoria, Australia

**Keywords:** fibrinogen, FIBTEM-A5, hemorrhage, prothrombin time, ROTEM, thromboelastography

## Abstract

**Background:**

Fibrinogen levels may fall early in major hemorrhage. The Clauss assay (Fib-C) is the laboratory gold standard, while the prothrombin time–derived assay (Fib-D) is a rapid, lower-cost alternative.

**Objectives:**

To compare Fib-C and Fib-D with viscoelastic-derived clot firmness.

**Methods:**

Data from all Queensland Health hospitals were extracted (2019-2025). Rotational Thromboelastometry FIBTEM-A5 was the reference standard. Paired Fib-C and Fib-D results with FIBTEM-A5 were analyzed. The primary outcome was correlation with FIBTEM-A5; secondary analyses assessed predictive performance for FIBTEM-A5 of ≤ 10 mm.

**Results:**

Of 2208 paired results were included, Fib-C correlated more strongly with FIBTEM-A5 than Fib-D (*r* = 0.85 vs 0.82; *P* = .008) and had higher accuracy for FIBTEM-A5 of ≤ 10 mm (area under the curve, 0.92 vs 0.90; *P* < .05). Optimal thresholds for FIBTEM-A5 of ≤ 10 mm were 2.1 g/L for Fib-C and 2.3g/L for Fib-D (*P* < .05).For absolute fibrinogen values, Fib-D produced higher mean levels than Fib-C (overall, 2.3 vs 2.1 g/L; *P* < .05), with differences observed in trauma (2.4 vs 2.2 g/L; *P* < .05) and chronic liver disease (1.9 vs 1.7 g/L; *P* < .05).

**Conclusion:**

Fib-C aligns more closely with viscoelastic clot firmness than Fib-D. These results support Fib-C as the preferred assay and discontinuing routine Fib-D reporting in critical bleeding.

## Essentials

• Clauss (Fib-C) and PT-derived fibrinogen (Fib-D) tests both measure fibrinogen, but the link between Fib-D and clot firmness has not been reported.
• This study compared Fib-C and Fib-D with ROTEM FIBTEM-A5 across Queensland Health hospitals.
• Fib-C matched viscoelastic clot firmness more closely than Fib-D.
• Results support Fib-C as the preferred test and stopping routine Fib-D reporting in critical bleeding.

## 1 Introduction

Fibrinogen plays an essential role in hemostasis and functions both as the precursor to fibrin, which is crosslinked to form blood clots, and as a mediator of platelet aggregation [1]. It circulates at the highest concentration of all coagulation proteins and is the first to fall to critically low levels during major hemorrhage [2]. Most major hemorrhage protocols (MHP) for nonpregnant adults recommend replacement of fibrinogen when levels are < 2.0 g/L [3].

There are several assays available to measure fibrinogen. The Claus assay (Fib-C) is a functional test based upon time for fibrin clot formation and it is considered the gold standard as it gives both qualitative and quantitative information. An alternative method is the prothrombin time (PT)-derived assay (Fib-D), which indirectly estimates fibrinogen concentration during the PT assay. Lastly, an immunologic assay is available to provide the most accurate quantitation and are largely only of value in investigating congenital dysfibrinogenemia [4].

Fib-D is a simple and inexpensive test and is widely used; however, it can give misleading results in some disorders and is not recommended for routine laboratory use [4]. Discrepancies have been observed between these Fib-C and Fib-D assays, and typically, Fib-D provides higher measures of fibrinogen than Fib-C. This is most common in contexts such as liver disease, disseminated intravascular coagulation, renal disease, and dysfibrinogenemia [5]. Proposed mechanisms include reliance on clotting curve assumptions rather than direct measurement and calibration with buffer-diluted plasma, which may alter the rate or mode of fibrin fibrils formation [6]. In laboratories where Fib-D is the standard assessment, action limits to trigger reflex testing of Fib-C are required to avoid reporting potentially inaccurate derived fibrinogen levels.

Viscoelastic hemostatic assays (VHAs) are a point of care test increasingly used to measure fibrin-based clot strength and inform transfusion decisions. Rotational thromboelastometry (ROTEM; Werfen), the most commonly used VHA in Queensland, Australia, includes fibrinogen-specific FIBTEM assays that isolate fibrin-based contribution to clot strength by pharmacologically suppressing platelet activity [7]. Strong correlations between VHA-based fibrinogen metrics and the Clauss assay have been reported [8]. The aim of this study was to evaluate the clinical concordance of Fib-C and Fib-D with VHA fibrin clot firmness, focusing on their suitability for identifying hypofibrinogenemia in critical bleeding when rapid fibrinogen replacement may be required.

## 2 Methods

### 2.1 Study design

This was a retrospective laboratory study of diagnostic accuracy including data from all Queensland Health hospitals between January 1, 2019, and April 15, 2025. Laboratory data were obtained from AUSLAB, the statewide laboratory information system, and linked with diagnostic codes from the Emergency Data Collection (EDC), and Queensland Hospital Admitted Patient Data Collection (QHAPDC). Data linkage methodology has been described previously [9]. The study is reported in accordance with Standards for Reporting of Diagnostic Accuracy (STARD) 2015 guidelines [10].

### 2.2 Reference and index tests

All patients with available ROTEM results were eligible for inclusion. FIBTEM-A5 (ROTEM) was the reference standard. The index tests were paired conventional coagulation test (CCT), where Fib-C and Fib-D were reported. Tests were considered paired if performed within 30 minutes, to account for geographical separation between testing sites [11]. Participants without available reference standard results were excluded.

### 2.3 Test methods

ROTEM testing was performed on ROTEM sigma. FIBTEM employed cytochalasedsin D in earlier assays, with the newer assay incorporating an additional platelet inhibitor, tirofiban.

Fib-C and Fib-D were measured on ACL TOP analyzers. Fib-C was measured using the von Clauss method with HemosIL QFA thrombin reagent. High-concentration bovine thrombin was added to diluted platelet-poor plasma, and clotting time, detected optically, was inversely proportional to fibrinogen concentration, calibrated against international plasma standards. Fib-D was calculated during the PT assay from changes in optical density, following addition of Thromborel S and calcium, with values interpolated from a calibration curve.

Impaired clot firmness was defined as FIBTEM-A5 of ≤ 10 mm by VHA. Hypofibrinogenemia was defined as fibrinogen < 2.0 g/L by CCT. These thresholds were selected in line with current MHPs [7].

Tests were categorized into the following groups: overall cohort, trauma, chronic liver disease (CLD), gastrointestinal bleeding, and obstetrics. Categorization was based on diagnostic codes from EDC and QHAPDC (Supplementary Material).

### 2.4 Measures of association

The primary measure of association was correlation of Fib-C or Fib-D with ROTEM FIBTEM-A5 and comparison of correlation coefficients. Secondary measures assessed the predictive performance of Fib-C and Fib-D for detecting impaired clot firmness (FIBTEM-A5 ≤ 10 mm).

### 2.5 Data analysis

Descriptive statistics were used to summarize patient and test characteristics and to compare Fib-C and Fib-D values, within the overall cohort and by clinical subgroups. Pearson correlation coefficients quantified associations between Fib-C or Fib-D and VHA parameters (FIBTEM-A5). Differences between correlation coefficients were tested using Fisher *r*-to-*z* transformation [12]. Logistic regression and receiver operating characteristic curves were used to evaluate the ability of Fib-C and Fib-D to classify FIBTEM-A5 of ≤ 10 mm; DeLong method and bootstrap resampling were used to calculate CIs and compare curve performance, and the Youden Index identified the optimal Fib-C or Fib-D threshold for detecting FIBTEM-A5 of ≤ 10 mm. Analyses were performed using R version 4.4.1 (R Foundation for Statistical Computing). A 2-sided *P* < .05 was considered statistically significant.

The study was approved by Metro South Human Research Ethics Committee (HREC/2025/QMS/116509). The requirement to seek informed consent from patients was waived.

## 3 Results

### 3.1 Study cohort

**Figure 1:**
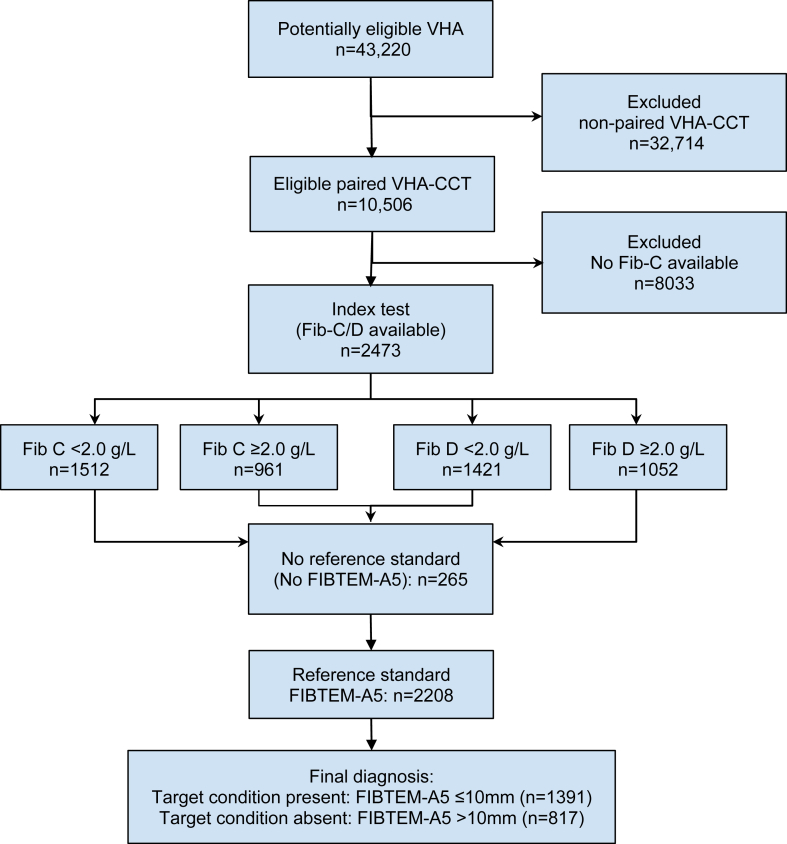
Analytical study flowchart and derivation of the final study cohort. Fibrinogen positive = level < 2.0 g/L; target condition present = FIBTEM-A5 ≤ 10 mm. CCT, conventional coagulation test; VHA, viscoelastic hemostatic assay.

During the study period, 43,220 VHAs were performed (39,776 ROTEM and 3444 thromboelastography [TEG]). Of the 10,506 paired VHA–CCT tests, 2473 had complete results for Fib-C and Fib-D. After excluding 265 cases where results of the reference standard were not available, 2208 tests were included in the analysis (Figure 1). Of these, 1391 (63%) had a FIBTEM-A5 of ≤ 10 mm. Baseline characteristics are presented in Table.

**Table:** Patient demographics and presentation characteristics.

Characteristic	Test results (*N* = 2208)
Unique patients (*n* = 1691)	
Age (y), mean (SD)	48 (20)
Gender	
Female	548 (32)
Male	1141 (68)
Missing	2
Unique presentations (*n* = 1814)	
Type of admission	
Elective admission	117 (12)
Emergency admission	771 (81)
Not assigned	65 (6.8)
Missing	861
Clinical subgroups	
Chronic liver disease	319 (18)
Obstetrics	72 (4.1)
Trauma	1149 (66)
Gastrointestinal bleed	290 (16)
Variceal bleed	71 (3.9)
Nonvariceal bleed	219 (12)

There were 1691 patients, accounting for 1814 presentations, with 2208 test results. Values are *n* (%) unless specified.

### 3.2 Correlation with VHA

**Figure 2:**
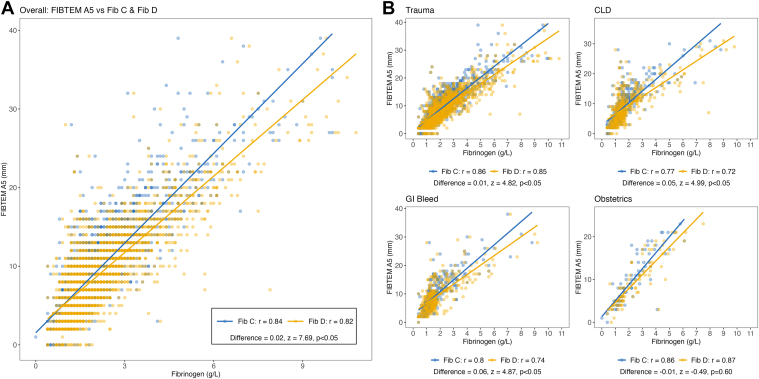
(A) Overall correlations between FIBTEM-A5 and Fib-C and Fib-D. (B) Correlations between FIBTEM-A5 and Fib-C and Fib-D by clinical diagnosis. GI, gastrointestinal.

Fib-C and Fib-D correlated strongly with FIBTEM-A5 (*r* = 0.85, *P* < .05; and *r* = 0.82, *P* < .05, respectively), with Fib-C showing a higher overall correlation (*z* = 7.69; *P* < .05) (Figure 2A). Correlations stratified by diagnosis are illustrated in Figure 2B. Among patients with trauma, CLD, and gastrointestinal bleed, Fib-C showed higher correlation with FIBTEM-A5 than Fib-D (all *P* < .05). For the obstetric subgroup, no differences were observed (*P* = .62) (Supplementary Table 1).

### 3.3 Predictive performance of Fib-C and Fib-D for FIBTEM-A5 of ≤10 mm

**Figure 3:**
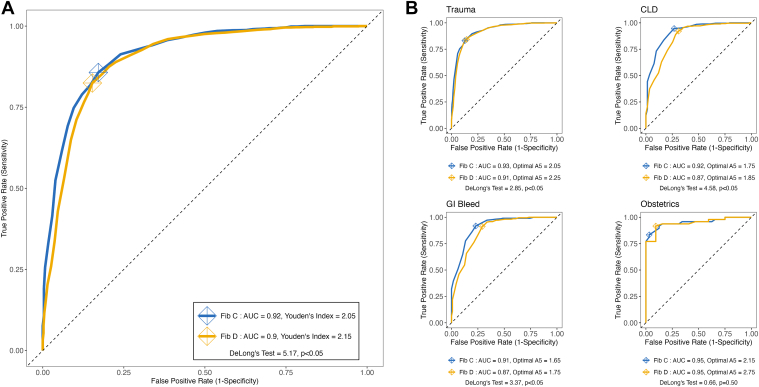
(A) Receiver operating characteristic (ROC) curves for Fib-C and Fib-D in detecting FIBTEM-A5 of ≤ 10 mm for overall cohort. (B) ROC for Fib-C and D in detecting FIBTEM-A5 of ≤ 10 or > 10 mm, by diagnosis. AUC, area under the curve.

Overall, Fib-C showed higher accuracy in predicting FIBTEM-A5 of ≤ 10 mm (area under the curve, 0.915; 95% CI, 0.903-0.927) than Fib-D (area under the curve, 0.900; 95% CI, 0.885-0.914; DeLong D, 5.17; *P* < .05) (Figure 3A). The optimal fibrinogen threshold for Fib-C was 2.1 g/L (sensitivity, 0.86; specificity, 0.83) and 2.2 g/L for Fib-D (sensitivity, 0.82; specificity, 0.85; *P* < .05). For diagnostic subgroups, Fib-C showed higher predictive performance in trauma, CLD, and gastrointestinal bleeding (all *P* < .05) (Figure 3B, Supplementary Table 2).

### 3.4 Fibrinogen levels by Fib-C and Fib-D

**Figure 4:**
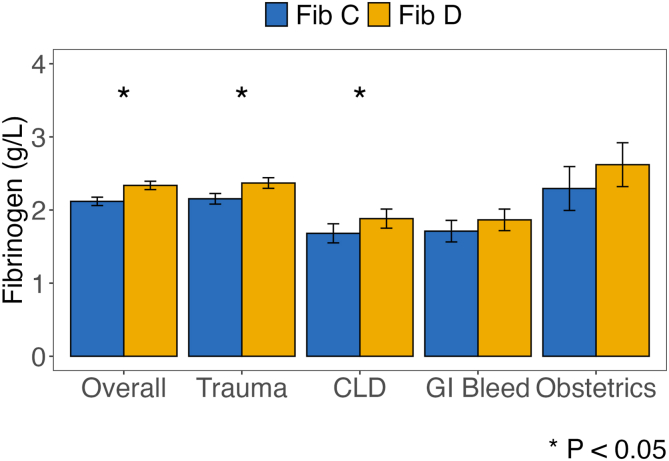
Mean fibrinogen (g/L) by Fib-C and Fib-D, with error bars denoting 95% CIs.

Mean fibrinogen was higher for Fib-D than for Fib-C in the overall cohort (2.3 vs 2.1 g/L; *P* < .05). By diagnosis, values were significantly higher for Fib-D in trauma (2.4 vs 2.2 g/L; *P* < .05) and in CLD (1.9 vs 1.7 g/L; *P* < .05) (Figure 4, Supplementary Table 3).

## 4 Discussion

Our study is the first to benchmark Clauss and PT-derived fibrinogen assays against VHA measures of clot firmness. The key finding was that both assays correlated strongly with fibrin-based clot firmness; however, Fib-C demonstrated consistently superior concordance and more accurate identification of clinically important thresholds for hypofibrinogenemia. This difference has meaningful clinical implications for the recognition of low fibrinogen levels and replacement.

In subgroup analyses, Fib-C more reliably identified patients with a low FIBTEM-A5 among patient subgroups of trauma, CLD, and gastrointestinal bleeding. The differences in the obstetric group did not reach statistical significance but may have been limited by low numbers. Among patients with CLD, hypofibrinogenemia, and dysfibrinogenemia are well documented in rebalanced hemostasis, and this qualitative and quantitative disturbances more pronounced in acute decompensation when disseminated intravascular coagulation is more common, which impacts assay performance [13,14]. Therefore, transfusion decisions based on Fib-C should be prioritized for patients with CLD where accurate fibrinogen assessment can inform periprocedural bleeding risk and guide transfusion decisions in acute bleeding.

In our study, the mean fibrinogen values were consistently higher for Fib-D than Fib-C in the overall cohort and statistically higher in trauma and CLD subgroups. Miesbach et al. [6] have reported up to a 5 times overestimation with PT-derived fibrinogen. For clinicians using MHPs that apply fixed fibrinogen thresholds to trigger fibrinogen replacement, such upward bias may meaningfully influence transfusion decisions. In contrast, Fib-C values showed closer numerical agreement with the functional assessment on VHA, potentially offering a more dependable measure of hemostatic capacity.

The receiver operating characteristic–derived optimal thresholds for detecting FIBTEM-A5 of ≤ 10 mm were 2.1 g/L for Fib-C and 2.3 g/L for Fib-D, values slightly above the commonly used 2.0 g/L trigger in MHPs. While the difference for Fib-C is small and broadly consistent with current practice, these findings indicate that impaired clot firmness, as defined by FIBTEM-A5 of ≤ 10 mm, may be observed at fibrinogen concentrations marginally > 2.0 g/L.

It is important to acknowledge some pragmatic strengths of the PT-derived assay including rapid turnaround time, lower reagent cost, and widespread availability. These attributes are favorable in laboratories with high throughput and/or limited resourcing across all domains. Our findings confirm that Fib-D retains acceptable correlation with FIBTEM-A5 when hemostasis is not profoundly disrupted. However, fibrinogen assessment in active hemorrhage requires high diagnostic fidelity; small differences in assay accuracy translate directly into earlier or delayed replacement. Our results support preferential use of Fib-C in any clinical scenario where fibrinogen replacement is being considered or where VHA are not available to directly quantify clot firmness.

In acute hemorrhage, turnaround time is central to clinical decision making. FIBTEM-A5 on ROTEM is available as early as 5 minutes as a point-of-care result and, therefore, precedes both Fib-C and Fib-D laboratory measurements in most settings [11]. In institutions where VHA is embedded within MHPs, transfusion decisions are commonly guided by early clot firmness rather than waiting for laboratory fibrinogen levels [7]. Accordingly, the intent of this study was not to position CCT against VHA but to determine which laboratory fibrinogen assay more closely reflects functional clot firmness when a laboratory value is required. In settings without VHA access or where fibrinogen concentration is needed for documentation, longitudinal monitoring, or protocolized triggers, alignment with functional hemostasis becomes particularly relevant [15].

This study has limitations inherent to retrospective research. A substantial proportion of patients were excluded because Fib-D was available without a corresponding Fib-C, introducing potential selection bias. The study did not account for the change from single to dual platelet inhibition in the FIBTEM assay, which may alter sensitivity to fibrin contribution [16]; its analytical impact is currently being formally evaluated by our group. The constraints of data linkage have previously been described [9]. TEG was not part of this analysis, and methodological differences between TEG and ROTEM—fixed-pin vs rotating-pin mechanics—may produce differing assessments of fibrin polymerization and clot strength [17]. There was minimal variance in laboratory coagulation testing as all Pathology Queensland laboratories use the same analyzers and reagents and perform testing in accordance with the National Association of Testing Authorities. On the contrary, ROTEM is a point of care test where intersite variability in quality control and compliance with sample requirements and test procedure cannot fully be accounted for, although these factors likely reflect real-world practice [11].

## 5 Conclusion

The Clauss method better detects low clot firmness than the PT-derived method, supporting more accurate fibrinogen replacement in the bleeding patient. These findings support standardizing Fib-C as the primary fibrinogen assay and discontinuing routine reporting of Fib-D in bleeding.
