# Supplementary material for: Diagnostic accuracy of Clauss and prothrombin time–derived fibrinogen against rotational thromboelastometry FIBTEM-A5
Source: Res Pract Thromb Haemost. 2026 Mar 26;10(3):103439. doi: 10.1016/j.rpth.2026.103439 (PMC13100266; doi:10.1016/j.rpth.2026.103439)
Supplement: Supplementary Material [file mmc1.docx]

### **SUPPLEMENTARY MATERIAL**

#### **Diagnosis by ICD-10-AM codes**

Trauma-related diagnoses were defined using ICD-10-AM codes S00–T98, which include injuries, poisoning, and other consequences of external causes^1^.

Chronic liver disease (CLD) was identified using a combination of ICD-10-AM codes for gastroesophageal varices with or without bleeding (I85.0, I85.9, I86.4, I98.3, I98.2), liver cell carcinoma (C22), alcohol-related liver disease (K70.0–K70.4, K70.9), toxic liver disease with fibrosis and cirrhosis (K71.7), hepatic failure (K72.1, K72.9), chronic hepatitis (K73.0–K73.2, K73.8, K73.9), fibrosis and cirrhosis (K74.0, K74.1, K74.3–K74.6), other inflammatory liver diseases (K75.2, K75.4, K75.8, K75.9), other liver diseases (K76.6, K76.8, K76.9), chronic liver failure (U843), hepatorenal syndrome (K76.6), and chronic viral hepatitis (B18). Ascites (R18) was included only if it was accompanied by one of the above CLD codes and/or any cirrhosis-related procedure codes (3047603, 3047602, 9033400, 3040600)^2^.

Variceal bleeding was defined by the presence of I98.3 or I85.0, while non-variceal Gastrointestinal bleeding was identified using K92 in the absence of variceal codes^2^.

Obstetric diagnoses were identified using ICD-10-AM codes O00–O99, which include all conditions related to pregnancy, childbirth, and puerperium^3^.

**TABLES**

Supplemental Table 1. Correlation of FIBTEM A5 with Clauss fibrinogen (Fib-C) and PT-derived fibrinogen (Fib-D), stratified by clinical diagnosis. P value denotes difference in Pearson’s r coefficients.

|  |  | Pearson's r | |  | |
| --- | --- | --- | --- | --- | --- |
| Diagnosis | N | Fib-C | Fib-D | z | P |
| Overall | 2208 | 0.85 | 0.82 | 7.69 | <0.001 |
| Trauma | 1366 | 0.86 | 0.85 | 4.82 | <0.001 |
| CLD | 422 | 0.77 | 0.72 | 5.00 | <0.001 |
| GI Bleed | 332 | 0.80 | 0.74 | 4.87 | <0.001 |
| Obstetrics | 80 | 0.86 | 0.87 | -0.49 | 0.623 |

CLD: Chronic Liver Disease, GI: Gastrointestinal.

Supplemental Table 2. Optimal threshold for predicting FIBTEM A5 ≤10mm or > 10 mm determined by Youden’s index, sensitivity, specificity and area under the curve (AUC) for each clinical diagnosis. DeLong’s D statistic tests for the difference between Fib C and D in terms of predictive performance (AUC) for FIBTEM A5 ≤10mm.

| Cohort | Fibrinogen | N | Threshold (g/L) | Sensitivity | Specificity | AUC | P value |
| --- | --- | --- | --- | --- | --- | --- | --- |
| Overall | Fib C | 2208 | 2.05 | 0.86 | 0.83 | 0.92 (0.90-0.93) | <0.001 |
|  | Fib D | 2208 | 2.15 | 0.82 | 0.85 | 0.90 (0.89-0.91) |  |
| Trauma | Fib C | 1366 | 2.05 | 0.83 | 0.87 | 0.93 (0.91-0.94) | <0.001 |
|  | Fib D | 1366 | 2.25 | 0.84 | 0.86 | 0.91 (0.90-0.93) |  |
| CLD | Fib C | 422 | 1.75 | 0.95 | 0.73 | 0.92 (0.89-0.94) | <0.001 |
|  | Fib D | 422 | 1.85 | 0.92 | 0.69 | 0.87 (0.83-0.91) |  |
| GI Bleed | Fib C | 332 | 1.65 | 0.92 | 0.77 | 0.91 (0.88-0.95) | <0.001 |
|  | Fib D | 332 | 1.75 | 0.91 | 0.71 | 0.87 (0.83-0.92) |  |
| Obstetrics | Fib C | 80 | 2.15 | 0.83 | 0.97 | 0.95 (0.91-0.99) | 0.50 |
|  | Fib D | 80 | 2.75 | 0.92 | 0.91 | 0.95 (0.90-0.99) |  |

Supplemental Table 3. Summary statistics of Fibrinogen levels by Fib-C and Fib-D. Independent samples Welch t-tests were used to compare means.

| **Variable** | **Fib C** **N = 2,208** | **Fib D** **N = 2,208** | **Welch t-test** **(df = Degrees of Freedom)** | **p-value** |
| --- | --- | --- | --- | --- |
| **Overall** |  |  | -5.2 (df = 4338.35) | <0.001 |
| Mean (SD) - g/L | 2.12 (1.30) | 2.34 (1.48) |  |  |
| **Trauma** |  |  | -4.07 (df = 2675.68) | <0.001 |
| Mean (SD) - g/L | 2.15 (1.28) | 2.37 (1.48) |  |  |
| **CLD** |  |  | -2.32 (df = 809.93) | 0.023 |
| Mean (SD) - g/L | 1.68 (1.13) | 1.88 (1.38) |  |  |
| **GI Bleed** |  |  | -1.61 (df = 649.89) | 0.11 |
| Mean (SD) - g/L | 1.71 (1.15) | 1.87 (1.31) |  |  |
| **Obstetrics** |  |  | -1.42 (df = 154.69) | 0.20 |
| Mean (SD) - g/L | 2.29 (1.34) | 2.62 (1.56) |  |  |

**REFERENCES**

1. Stephenson S LJ, Henley G, Harrison JE.,. Diagnosis-based injury severity scaling: A method using Australian and New Zealand hospital data coded to ICD-10-AM. Canberra: Australian Institute of Health and Welfare; 2003.

2. Powell EE, Skoien R, Rahman T, Clark PJ, O'Beirne J, Hartel G, et al. Increasing Hospitalization Rates for Cirrhosis: Overrepresentation of Disadvantaged Australians. EClinicalMedicine. 2019;11:44-53.

3. WA Clinical Coding Authority. Guide to Major Eleventh Edition Changes: Obstetrics, neonates and genitourinary. Perth, WA; 2020.
